# Inverse correlation between urethral length and continence before and after native tissue pelvic floor reconstruction

**DOI:** 10.1038/s41598-021-01565-z

**Published:** 2021-11-10

**Authors:** A. R. Mothes, H. K. Mothes, A. Kather, A. Altendorf-Hofmann, M. P. Radosa, J. C. Radosa, I. B. Runnebaum

**Affiliations:** 1 Women’s University Hospital of Jena, Department of Gynaecology and Reproductive Medicine, Jena University Hospital, Friedrich-Schiller-University Jena, Am Klinikum 1, 07747 Jena, Germany; 2 Present Address: Department of Gynaecology, St. Georg Hospital Eisenach, Academic Teaching Hospital of University of Jena, Eisenach, Germany; 3 Department of General, Visceral and Vascular Surgery, Jena University Hospital, Jena, Germany; 4 Present Address: Department of Abdominal and Vascular Surgery, Sophien and Hufeland Hospital Weimar, Academic Teaching Hospital of University of Jena, Weimar, Germany; 5 Present Address: Department of Gynaecology and Obstetrics, Hospital Bremen North, Bremen, Germany; 6 Department of Obstetrics & Gynaecology, Saarland University Hospital, Homburg/Saar, Germany

**Keywords:** Urinary incontinence, Urethra

## Abstract

Urethral length was evaluated retrospectively in patients with prolapse undergoing anterior native-tissue repair. Effects of age, prolapse stage, defect pattern, urodynamic and clinical stress test findings, and tension-free vaginal tape (TVT) surgery indication were analyzed using Mann–Whitney and Wilcoxon tests and linear and logistic regression. Of 394 patients, 61% had stage II/III and 39% had stage IV prolapse; 90% of defects were central (10% were lateral). Median pre- and postoperative urethral lengths were 14 and 22 mm (*p* < 0.01). Preoperative urethral length was greater with lateral defects [*p* < 0.01, *B* 6.38, 95% confidence interval (CI) 4.67–8.08] and increased stress incontinence risk (*p* < 0.01, odds ratio 1.07, 95% CI 1.03–1.12). Postoperative urethral length depended on prolapse stage (*p* < 0.01, *B* 1.61, 95% CI 0.85–2.38) and defect type (*p* = 0.02, *B* – 1.42, 95% CI – 2.65 to – 0.2). Postoperatively, TVT surgery was indicated in 5.1% of patients (median 9 months), who had longer urethras than those without this indication (*p* = 0.043). Native-tissue prolapse repair including Kelly plication increased urethral length, reflecting re-urethralization, particularly with central defects. The functional impact of urethral length in the context of connective tissue aging should be examined further.

## Introduction

The reported lifetime risk of primary surgery for stress urinary incontinence (SUI) or pelvic organ prolapse (POP) is 20%^1^, but whether continence surgery should be combined with or be secondary to prolapse repair after follow up remains unclear^2^. Sling procedures are considered to be the gold standard for SUI surgery, but they usually require the use of alloplastic materials, which is associated with disadvantages and potential complications^3^, like voiding dysfunction, pain, vaginal tape erosion or exposure, vaginal or bladder perforation, vascular or visceral injury as well as dislocation and failure at long term subjective cure rates ranging from 43 to 92%^4^. In this context, it is important to discuss advantages and risks of concurrent sling procedures with patients^5–8^. The question whether patients with POP combined with SUI preoperatively or developing de novo SUI postoperatively would benefit from simultaneous sling procedures or not has not been scientifically answered yet^9^. The maintenance or achievement of urinary continence after native-tissue prolapse repair is of clinical importance to avoid the need for continence procedures with the simultaneous use of alloplastic grafts in as many patients and for as long as possible.

Prospective randomized controlled trials have shown that anterior native-tissue repair for POP leads to lower de-novo SUI rates than the use of vaginal armed mesh implants does^2^. Thus, further investigation and the development of techniques for site-specific anterior native-tissue repair with Kelly plication^10–14^ to provide additional urethral support in combination with central or lateral vaginal repair would be of value. Although the aim of pelvic floor surgery is to restore the anatomy, we hypothesize that anatomical correction with reconstructive surgery would result in the improvement of function. Additional to anatomical fascia defects, that are approached by reconstructive surgery, aging and connective tissue weakness are considered to be risk factors contributing to pelvic floor disorders^2^. Therefore, and in the context of anatomical reconstruction, suburethral support using native tissue (as in Kelly plication) or alloplastic implants (as in sling procedures) might be as important as restoration of anatomy for the improvement of function. Other questions that need to be addressed include whether the anatomic urethral length changes following female native-tissue prolapse repair, whether changes in urethral length (postoperative *vs*. preoperative) differ by preoperative prolapse stage and/or anterior defect pattern, and what relative effects anatomic urethral length has on the urethral continence mechanism.

The aim of this study was to evaluate intraoperative anatomic urethral lengths, measured between the internal and external urethral orifices, in patients undergoing anterior vaginal native-tissue repair for POP including Kelly plication, midline colporrhaphy for central anterior vaginal defects, vaginal paravaginal repair of lateral defects, and concomitant apical repair. A secondary aim was to compare data according to prolapse stage (II/III *vs*. IV) and anterior compartment defect location (central *vs*. lateral). In anterior compartment prolapse, bladder neck and urethral descent are frequently found on clinical examination. According to recent ultrasound findings, urethral length shortening and urethral funneling reflect the dynamic behaviour of bladder neck and proximal urethra as revealed by dynamic ultrasound assessments^15^. Anatomical bladder neck reconstruction including the correction of urethral funneling is expected to result in a re-urethralisation of the proximal urethra. Therefore, we hypothesized that the urethral length would be greater than preoperatively after reconstructive anterior compartment native-tissue surgery including Kelly plication. Clinical and ultrasound investigations^16^ of urethral funneling revealed the phenomenon to be either associated with central or with lateral fascial defects. The pathogenesis of urethral funneling as well as its association with fascial defects remains unclear^17^, but tissue weakness leading to impaired urethral tonus might be one of the underlying factors. According to MRI investigations of urethral anatomy by DeLancey’s group^18^, urethral tonus is provided by the urethral smooth muscles, the urethral striated muscle, and the vascularisation within the submucosa. Atrophy of these structures have been shown to be associated with urethral funneling and incontinence^19–21^. Due to the technical nature of Kelly plication giving sub-urethral support rather than closing lateral defects, we hypothesized that the urethral length would be greater than preoperatively with central than with lateral defects. The severity of anterior vaginal wall prolapse was correlated with urethral function as observed by urodynamic investigations^22^. The extent of anatomical changes in anterior vaginal wall prolapse might have an influence on anatomical urethral length change. Therefore, we hypothesized that the urethral length would be greater than preoperatively with advanced (stage IV) than with stage II/III prolapse. Another aim of this study was to evaluate the influence of urethral length on continence, as reflected by preoperative incontinence symptoms, intrinsic sphincter deficiency (ISD), and the postoperative need for tension-free vaginal tape (TVT) procedures. Urethral funneling has been shown to be anatomically associated with shortened urethras as well as with urinary incontinence in up to 97%^17^. Other groups found a positive correlation between anatomical urethral length and urethral closure^23^. Therefore, we hypothesized shorter urethras to be associated with ISD and preoperative incontinence symptoms, and that longer post-repair urethras would be associated with better postoperative continence. This basic analysis is meant to contribute to the discussion of the roles of urethral length and stabilization of the vesico-urethral junction in the urethral continence mechanism.

## Methods

### Design and sample

This single-center retrospective observational study was conducted with data from patients with POP who had undergone vaginal native-tissue repair of central or lateral anterior-compartment defects at the Pelvic Floor Center of Jena University Hospital, Germany. Consecutive cases were included based on the availability of urethral length data in surgical reports between May 2010 and August 2014. Included patient´s clinical examination and surgical exploration revealed bladder neck and urethral descent. All patients presented multi-compartment defects. Patients presenting a history of previous urethral surgery, pelvic radiation, spinal cord stenosis, other neurogenic bladder dysfunction, large pelvic tumors, urogenital malformations, as well as the urodynamic findings of detrusor overactivity, bladder outlet obstruction, detrusor sphincter dyssynergia, and urinary tract infections at the time of surgery were excluded.

The study was conducted in accordance with the ethical standards of the Declaration of Helsinki, and was approved by the General Ethics Commission of the Faculty of Medicine, Jena University Hospital (protocol no. 2019-1272). All patients provided written informed consent prior to examinations and surgeries.

### Data collection

Data on patient age, prolapse stage, anterior compartment defect pattern, concomitant apical repair, preoperative SUI symptoms, preoperative ISD (obtained by urodynamic study), clinical stress test results, indications for TVT surgery according to quality of life (QoL) impairment, and the interval between prolapse repair and TVT surgery were extracted from hand-written and electronic patient files. Telephone interviews were conducted to obtain missing follow-up information on post-prolapse repair SUI symptoms, on the subjective need for further advice on SUI treatment and on eventual presentation to external pelvic floor centres in the meantime*.*

### Definitions and measurements

SUI was defined as “the complaint of any involuntary loss of urine on effort or physical exertion (e.g. sporting activities) or on sneezing or coughing”^24^ according to the International Continence Society (ICS) definition.

ISD was defined as urine leakage with increased abdominal pressure in the absence of detrusor contraction at the time of leakage, according to Abrams et al.^25^ and with awareness of the controversy concerning the definition of ISD^26^. Briefly, urodynamic measurements were performed by the same examiner in a standardized manner in our institution. The patient is placed on a half upright position and prolapse reposition is performed using a swab. Initially the pressure of the system must be zeroed and the transducer has to be maintained at the level of the pubic bone. Bladder filling is performed at 60 ml/min until obtaining a volume of 300 ml. Maximal urethral closure pressure (MUCP) is measured at rest and the abdominal leak point pressure (ALPP) is measured at repeated Valsalva maneuver or coughing. When interpreting the curves, MUCP of < 20 cm H_2_O and ALPP of 60 cm H_2_O were used as parameters to obtain ISD diagnosis. Standardized cough tests were performed on a routine base with prolapse reposition in half upright, sitting and standing position before patients were ask to empty the bladder and urine flow rates were taken. Ingelman-Sundberg scale was used to classify SUI^27^. Anterior compartment defects were determined to be central or lateral by clinical vaginal examination according to the vaginal rugae, as well as during surgical tissue preparation^28–30^.

**Figure 1:**
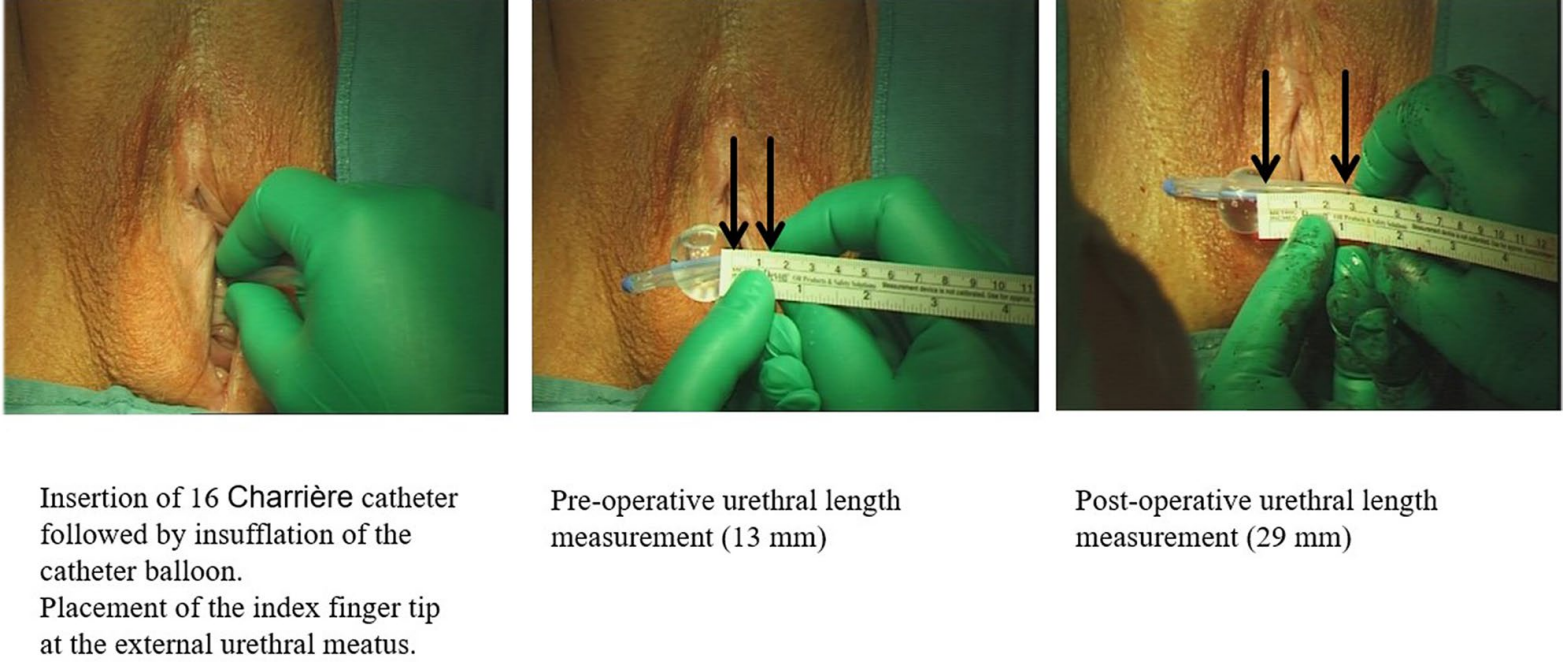
Catheter method for anatomical urethral length measurements.

At the beginning and end of each defect repair procedure, the location of the vesico-urethral junction was identified and the anatomic urethral length was determined using the catheter method, described elsewhere^31^. Briefly, a 16-Charrière Foley catheter was inserted transurethrally, followed by insufflation of the catheter balloon and placement of the operator’s index fingertip under slight traction at the site of the external urethral meatus. After deflation of the balloon, the catheter was removed and inflated again externally. The measurement was taken from the balloon to index fingertip, as shown in Fig. 1.

### Surgical procedures

One experienced surgeon certified for pelvic floor reconstructive surgery by the German Society of Obstetrics and Gynaecology (level III) performed all surgeries and examinations included in this study. Defect-specific anterior colporrhaphy for central and lateral anterior vaginal defects^32^, and Kelly plication of fascial tissues at the vesico-urethral angle for urethral support^10–12^, were performed using traditional techniques. These procedures were combined with posterior and/or apical compartment repair, depending on the defect pattern. For Kelly plication, the bladder neck and proximal urethra were identified by clinical examination, a midline incision was made, and the vaginal epithelium was dissected and separated from the bladder by cutting the fascia fibrils with a focus on the bladder neck and proximal urethra. Mattress sutures (PDS 2-0, Ethicon; Johnson & Johnson International) were used to bring together the available connective tissue around the bladder neck^11^. Especially in patients with short urethras, the tissue around the bladder neck is frequently relaxed and soft. The placement of suburethral plication sutures in the surrounding tissue elevates the bladder neck and supports it with the connective fascial tissue and pubo-urethral ligaments^11^. This technique allows tightening of the proximal urethra and its inclusion in the urethral canal, and is termed “re-urethralization.”

### Pre- and postoperative care

Preoperatively, patients’ urogynaecological histories were taken using questions according to the validated German pelvic floor questionnaire^33^ and according to the Ingelman-Sundberg scale^27^ in a patient-centered approach. Patients were asked whether urine leakage occurs with suddenly raised abdominal pressure like in coughing, sneezing, laughing or heavy lifting, while running, walking, climbing stairs, or while getting up from a sitting position. Treatment decisions were made according to patients’ histories and reported QoL impairment. Patients were counselled about the phenomenon of hidden incontinence prior to prolapse surgery to prevent disappointment in cases of postoperative incontinence.

Routine postoperative management strategies included follow-up visits at 1 week and 3 months after prolapse surgery. At the 3-month evaluation, patients’ interval histories were taken with a focus on incontinence, and further treatment options and the risks and benefits of alloplastic grafts were discussed. Patients with symptoms of incontinence were asked to subjectively judge their QoL impairment before they chose conservative treatment or TVT surgery.

### Statistical analysis

The SPSS software package (IBM SPSS Statistics for Windows, version 27.0; IBM Corporation, Armonk, NY, USA) was used for statistical analysis. Categorical variables are reported as percentage and for continuous variables median and range are presented. Shapiro–Wilk test indicated that data did not follow a normal distribution. Therefore, comparisons between groups for continuous variables were performed using Mann–Whitney-U-test. Wilcoxon signed-rank test was used for comparison of paired groups. Effects of study factors on pre- and postoperative urethral length were analysed by multivariate linear regression analysis. Logistic regression models were used to examine influence of study factors on risk for preoperative SUI with and without adjustment for ISD.

## Results

### Baseline characteristics

The sample comprised 394 patients with a median age of 66 (range, 33–89) years; 149 (38%) patients were older than 70 years old. Telephone interviews were conducted with 68 patients to obtain missing follow-up information. In total, 239 (61.3%) patients presented with stages II and III prolapse, and 155 (38.7%) patients had stage IV prolapse. Intraoperative examination during surgical preparation revealed that anterior compartment defects were central in 355 (90.1%) patients and lateral in 39 (9.9%) patients (Table 1). Apical defect repair was performed in 186 (47%) patients.

**Table 1:** Baseline characteristics of the study sample (*n* = 394). SUI, stress urinary incontinence.

Characteristic	Median (range) or *n* (%)
Age (years)	66 (33–89)
Prolapse grade II/III	239 (61.27%)
Prolapse grade IV	155 (38.73%)
Central anterior defect	355 (90.1%)
Lateral anterior defect	39 (9.9%)
Preoperative SUI symptoms	167 (42.4%)
Preoperative urethral length (mm)	14 (3–38)
Postoperative urethral length (mm)	22 (12–49)

### Pre- and postoperative urethral lengths

**Figure 2:**
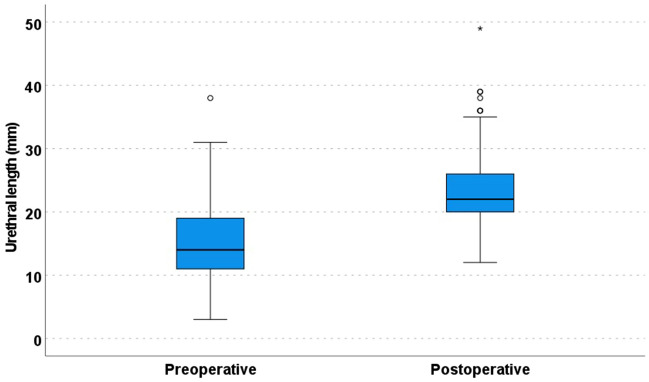
Boxplot of pre- and postoperative urethral length (*p* < 0.01, Wilcoxon signed-rank test).

The median preoperative and postoperative urethral lengths were 14 (range, 3–38) mm and 22 (range, 12–49) mm, respectively (*p* < 0.01, Wilcoxon signed-rank test; Table 1, Fig. 2). The median difference between these lengths was 8 (range, 0–29) mm.

Preoperatively, median urethral length was 14 (range, 3–31) mm in patients with prolapse stage IV and 15 (range, 5–38) mm in patients presenting with stage II/III (*p* = 0.426, Mann–Whitney-U-Test). Concerning defect type, in patients with lateral anterior defects preoperative urethra length (median, 21 mm; range, 7–38 mm) was longer (*p* < 0.01, Mann–Whitney-U-Test) compared to patients with central anterior defects (median, 14 mm; range 3–30 mm). In patients, which needed apical repair during POP surgery, median preoperative urethra length was 16 (range, 5–38) mm compared to 13 (range, 3–27) mm in patients without apical repair (*p* < 0.01, Mann–Whitney-U-Test).

The effect of the interdependent study factors on preoperative urethra length was studied using multivariate linear regression analysis (Table 2). Patient age (*B* 0.001, 95% CI – 0.05 to 0.05, *p* = 0.98) and prolapse stage (*B* 0.22, 95% CI − 0.83 to 1.27, *p* = 0.68) did not influence preoperative urethral length, whereas lateral anterior defect represented a factor, which was associated with increased length of urethra in POP patients (*B* 6.38, 95% CI 4.67 to 8.08, *p* < 0.01).

**Table 2:** Effects of study factors on preoperative urethral length, as determined by multivariate linear regression analysis.

Variable	B (95% CI)	*p*	EMM (standard error)
Age (years)	0.001 (–0.05 to 0.05)	0.98	n/a
Prolapse (stage IV vs. II/III)	0.22 (–0.83 to 1.27)	0.68	17.81 (0.57) vs. 17.58 (0.46)
Defect (lateral vs. central)	6.38 (4.67 to 8.08)	< 0.01	20.88 (0.83) vs. 14.51 (0.27)

B, regression coefficient; CI, confidence interval; EMM, estimated marginal mean.

After anterior native-tissue repair, patients with prolapse stage IV had a median urethra length of 24 (range, 14–49) mm and patients with stage II/III of 22 (range, 12–39) mm (*p* < 0.01, Mann–Whitney-U-Test). In patients with lateral anterior defects, median postoperative urethra length was 24 (range, 15–39) mm, while in patients with central anterior defects, urethra length was 22 (range, 12–49) mm (*p* = 0.014, Mann–Whitney-U-Test). If apical repair was performed during surgery, median postoperative urethra length was 25 (range, 14–49) mm compared to 21 (range, 12–35) mm in patients without apical repair (*p* < 0.01, Mann–Whitney-U-Test).

In order to unravel the influence of the different study factors on changes of urethral length due to anterior native tissue repair surgery, a multivariate linear regression analysis controlling for age and preoperative urethral length (Table 3) was performed. Magnitude of change in urethral length depended on the prolapse stage (*p* < 0.01) and defect type (*p* = 0.02), while apical repair did not influence the extent of increase in urethral length (*p* = 0.09). For patients with stage IV prolapse, increase in urethra length after surgery was higher compared to patients with stage II/III prolapse (*B* 1.61, 95% CI 0.85 to 2.38) Concerning patients with lateral anterior defects, the effect of surgery on lengthening of the urethra was lesser compared to patients with central anterior defects (*B* – 1.42, 95% CI – 2.65 to – 0.2).

**Table 3:** Effects of study factors on postoperative urethral length, as determined by multivariate linear regression analysis adjusted for age and preoperative urethral length.

Variable	B (95% CI)	*p*	EMM (standard error)
Prolapse (stage IV vs. II/III)	1.61 (0.85 to 2.38)	< 0.01	23.89 (0.4) vs. 22.28 (0.33)
Defect (lateral vs. central)	–1.42 (–2.65 to –0.2)	0.02	22.38 (0.59) vs. 23.8 (0.18)
Apical repair (yes vs. no)	0.66 (–0.09 to 1.41)	0.09	23.41 (0.37) vs. 22.76 (0.36)

B, regression coefficient; CI, confidence interval; EMM, estimated marginal mean.

### Preoperative function

Symptoms of SUI were reported in 167 (42.4%) patients preoperatively. Univariate binary logistic regression analysis showed that preoperative SUI was associated with patient age [odds ratio (OR), 0.95; 95% confidence interval (CI), 0.93–0.97; *p* < 0.01], prolapse stage (OR, 0.31; 95% CI 0.2–0.48; *p* < 0.01), and urethral length (OR, 1.06; 95% CI 1.02–1.1; *p* < 0.01; Table 4). More advanced age and prolapse decreased the risk of SUI, whereas longer urethras increased this risk. This was also observed if multivariate binary regression analysis including all study factors was performed (Table 4), with only minor differences in values of odds ratios and statistical significance. Concerning defect type, reduction of risk for patients with lateral anterior defects compared to patients with central anterior defects was only observed in multivariate binary logistic regression analysis controlling for all other study factors (OR, 0.42; 95% CI 0.19–0.92; *p* = 0.03; Table 4), but not in univariate analysis (OR, 1.19; 95% CI 0.61–2.3; *p* = 0.62; Table 4).

**Table 4:** Factors affecting the risk of stress-induced urinary incontinence in patients with pelvic organ prolapse, as determined by univariate and multivariate binary logistic regression analysis, respectively.

Variable	Univariate		Multivariate		Multivariate adjusted for ISD	
	OR (95% CI)	*p*	OR (95% CI)	*p*	OR (95% CI)	*p*
Age (years)	0.95 (0.93 to 0.97)	< 0.01	0.95 (0.93 to 0.97)	< 0.01	0.95 (0.92 to 0.98)	< 0.01
Pre-repair urethral length (mm)	1.06 (1.02 to 1.1)	< 0.01	1.07 (1.03 to 1.12)	< 0.01	1.07 (1.01 to 1.14)	0.02
Prolapse (stage IV vs. II/III)	0.31 (0.2 to 0.48)	< 0.01	0.33 (0.21 to 0.53)	< 0.01	0.32 (0.16 to 0.64)	< 0.01
Defect (lateral vs. central)	1.19 (0.61 to 2.3)	0.616	0.42 (0.19 to 0.92)	0.03	0.28 (0.1 to 0.8)	0.02

The multivariate analysis was in addition adjusted for intrinsic sphincter deficiency (OR, 7.75; 95% CI 3.87–15.5; *p* < 0.01). OR, odds ratio; CI, confidence interval.

Data on preoperative urodynamic measurements with prolapse repositioning were available for 235 patients. In 148 cases, these studies revealed preoperative ISD and positive results of clinical stress tests. Of the patients with preoperative ISD, 63% had symptoms of SUI; in contrast, 17% of the 87 patients without preoperative ISD had SUI symptoms. Adjustment of the binary logistic regression analysis of SUI risk for ISD did not substantially change the results (Table 4).

### Postoperative need for TVT surgery

**Figure 3:**
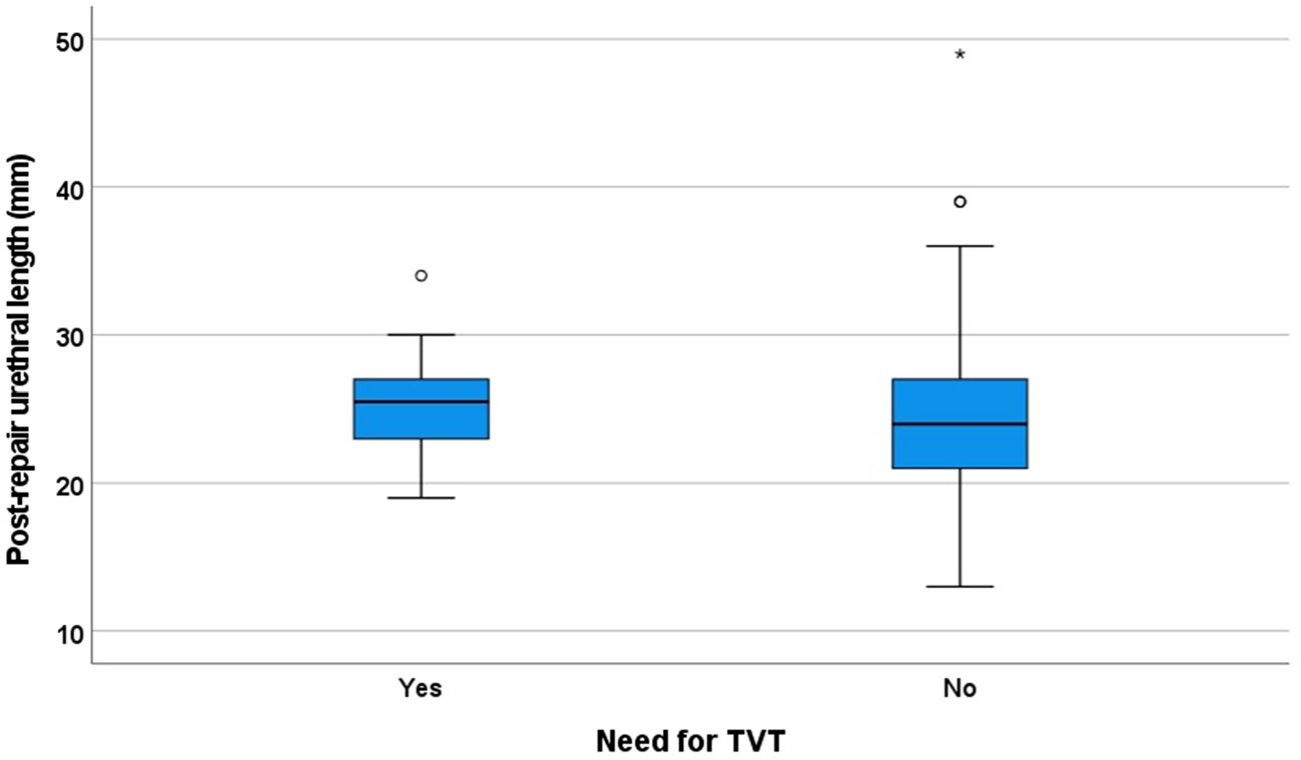
Boxplot of postoperative urethral lengths according to the need for TVT procedure (*p* = 0.043, Mann–Whitney-*U*-test). TVT, tension-free vaginal tape.

Follow-up data were available for all 394 patients. The median follow-up duration was 12 (range, 3–50) months. Of the 167 patients who reported preoperative SUI symptoms, 153 (92%) patients had no such symptom after prolapse surgery. Of the 227 patients without preoperative SUI symptoms, 6 (3%) reported de-novo symptoms of this condition after prolapse surgery and wished to undergo TVT procedures. Thus, a total of 20 (5.1%) patients had postoperative SUI requiring TVT procedures. The median interval between prolapse surgery and TVT procedures was 9 (range, 4–24) months. Median postoperative urethral lengths in patients without and with the need for TVT procedures were 22 (range, 12–49) mm and 26 (range, 16–34) mm, respectively (*p* = 0.043, Mann–Whitney- *U*-test; Fig. 3).

## Discussion

This study was performed to examine anatomic urethral lengths measured using a simple catheter method before and after anterior vaginal repair for POP in a large patient cohort, with analysis of their associations with pre- and postoperative parameters and functional outcomes. Anatomic urethral lengths were greater than preoperatively after site-specific anterior native-tissue repair with Kelly plication to provide additional suburethral support. Our results provide evidence that urethral length would increase after such surgical repair with re-urethralization. Preoperative urethral lengths were greater in patients with lateral defects. However, age and prolapse stage showed no association with urethral length before surgery. Increase in urethra length due to surgery was greater in patients with stage IV POP and in those with central defects, while apical repair showed no effect. The risk of SUI was greater in patients with less-advanced POP and in those with longer preoperative urethras. A small proportion of patients wished to undergo TVT procedures postoperatively. The need for TVT surgery was associated with greater postoperative urethral length. The study cohort comprised patients of typical age with a typical proportion of central anterior defects, but a high proportion of stage IV prolapse.

Surgical reconstruction was more effective, providing greater urethral support, in patients with central defects than in those with lateral defects in this study. The Kelly plication technique seems to have been developed for central defects; when it was first described, the defect type was not differentiated^10–13^. In our clinical experience, many lateral defects present with minimal fascial tissue available for suturing, which likely contributes to this difference in effectiveness. Additionally, it has to be discussed that rotational urethral descend often found in lateral defects is not frequently related to extensive urethral funneling and therefore to shortened urethras^34,35^. Less urethral shortening is obviously found in cases of slight funneling or in cases of a maintained urethro-vesical angle. Our study finding of preoperative greater urethral lengths in patients with lateral defects has to be interpreted accordingly. However, Goldammer found no difference in urethral funneling rate comparing central and lateral fascial defects^16^. Furthermore, study results confirm our hypothesis that urethral length would be greater than preoperatively with stage IV prolapse repair compared to stage II/III prolapse. The finding could be related to the effectiveness of Kelly plication in bladder neck reconstruction and in large prolapse disease.

Our approach to native-tissue surgery follows the concept that anatomical reconstruction leads to functional restoration, but our data do not support the hypothesis that longer urethras would be associated with better continence. Not only the anatomy, but also the tissue quality, appears to contribute to pelvic floor dysfunction. Despite other groups reported longer urethras to be correlated with better continence^16,17,23^, our study data do not support this findings. There are a few studies available reporting longer urethras rather being associated with incontinence^36,37^. Najjari et al. demonstrated longer urethras in incontinent patients performing perineal sonography^36^. Kupec et al. found greater urethral length and wider urethral lumen in SUI patients compared with healthy controls^37^. Obviously, other factors than pure anatomical length contributed to physiological urethral function. DeLancey^38^ and Dietz^39^ found elongated urethras in SUI patients to be correlated with pelvic floor insufficiency, vaginal deliveries and age. As connective tissue weakness is a risk factor for vaginal prolapse, it was assumed that impaired urethral tonus caused by weakness and atrophy of urethral and peri-urethral tissue could cause anatomically longer urethras^19–21^. ISD is believed to be caused by urethral and periurethral tissue weakness, which results in sphincteric mechanism deficiency. Such weakness can result from age, childbirth, and menopause. Our study results suggest that urethral stability contributed more than urethral length to the continence achieved after Kelly plication.

Normal female urethras are known to be longer than 30 mm. Lesser preoperative lengths, as observed in anterior compartment prolapse with catheter method assessment, could be caused by soft-tissue traction (e.g., due to immanent connective-tissue weakness in patients with prolapse) and bladder neck funneling, usually reflected by the finding of an urethrocele. Suburethral plication sutures, such as Kelly sutures, not only elevate, but also support, the bladder neck. Hypothetically, increased urethral length reflects anatomically correct surgical fascia reconstruction. Although not part of this study, we observed such outcomes by perineal ultrasound after prolapse surgery; a prospective magnetic resonance imaging (MRI) or ultrasound study should be conducted to confirmation our observation. Contrary to our hypothesis, concomitant apical repair did not influence urethral length in the present study cohort. Whether tissue traction and tightening stabilize the anterior vaginal wall, leading to postoperatively longer urethras, should be investigated prospectively. Low de-novo SUI rates after apical native tissue repair have been reported by others^40^.

The use of vaginal alloplastic grafts only when patients are symptomatic and desire this procedure, as well as in situations of POP recurrence, is a reasonable approach^3^. Limited lifetime exposure to vaginal alloplastic implants reduces the risk of complications that can impact QoL. Thus, our group investigates factors that may contribute to the achievement of more sustainable results of native-tissue POP repair using non-invasive, low-cost, and user-friendly methods, such as the catheter method, for the determination of vesico-urethral junction location and urethral length measurement. The proportion of patients needing TVT surgery was smaller than those reported in the literature. It may reflect the stabilizing effect of the performance of Kelly plication to establish suburethral support. Safety communication on synthetic mesh material used in pelvic floor surgery might also have contributed to patient´s hesitation seeking TVT surgery. Although 2011 FDA^3^ warning concerned only transvaginal mesh implants for prolapse, there was a drastic decline in TVT use observed following the FDA communication^41^. We examined the post–prolapse repair need for TVT surgery as a functional outcome rather than global postoperative incontinence, which often requires only conservative treatment within the evaluation interval of this study.

When counseling patients, questions about surgery to simultaneously treat prolapse and incontinence, and those about hidden incontinence, arise. For this reason, institutional treatment results such as the need for TVT surgery after prolapse repair can facilitate patient-centered counseling before prolapse surgery.

Surgical treatment of the anterior compartment can be technically challenging because it often requires accurate analysis and correction of different fascial defect patterns or combinations of anterior support defects^32^. Endopelvic fascial defects should be assessed carefully during intraoperative examination performed by an experienced surgeon^1^. MRI as being more reliable for diagnosing fascial defect locations might not be immediately available to answer questions during surgery. Anyway, the approach might be different from school to school. In our centre´s routine, diagnosis mainly rely on clinical examinations and on surgical exploration additionally to ultrasound. Sensitivity and specificity of vaginal examination or surgical exploration diagnosing lateral defects ranged from 24 to 94% and from 50 to 80% accordingly^28–30^. The wide range given in the literature indicates the examiners experiences playing a central role in diagnosing fascial defect pattern.

Kelly and Dumm^10^ combined anterior cystocele repair with plication of the tissue at the bladder neck, a simple procedure with immediate and long-term success rates of 90% and 65%, respectively. Morphological examination of urethral defects using MRI has revealed reduced posterior tissue layers in 37% of patients and dehiscence of the posterior muscle layer in an additional 13% of patients with proven SUI^28^. Thus, a surgical technique that provides support for the posterior urethral region at the vesico-urethral junction using the native tissue should improve urethral function and continence. The influence of vaginal anterior native-tissue repair for female genital prolapse with Kelly plication on the re-urethralization of the vesico-urethral junction, and thereby on the anatomic urethral length, has not been examined previously.

Urethral mobility and the correction of the vesico-urethral angle during anterior repair in native-tissue prolapse surgery play obvious roles in the urinary continence mechanism^42,43^ and might explain the findings of this study concerning the functional impact of anatomically determined urethral length. However, the effect of vesico-urethral junction re-urethralization during POP surgery on the urethral continence mechanism remains unclear; imaging and functional studies of this effect are needed.

The results of this study provide basic information on changes in anatomic urethral length and its possible functional impact on continence after anterior native-tissue repair with Kelly plication, which may serve as a basis for further research. Especially in the context of international discussions about the use of alloplastic grafts in pelvic floor surgery, our group’s clinical research focuses on surgical native-tissue repair techniques. However, the study has some limitations. According to Hosker^44^, ISD is an “imprecise diagnosis” due to poorly standardized urodynamic measurements. There seems to be no consensus amongst urologists and urogynaecologists on how to exactly perform these measurements and on how to interpret results leading to the diagnosis of ISD. The urodynamic data were incomplete and the examiner’s intraoperative identification of vaginal anterior defect patterns was subjective, based on experience rather than on more-objective diagnostic tools. Furthermore, for robust statistical evaluation of the influence of the defect type on urethra length and continence, the number of patients with lateral anterior defect might be too small. Preoperative imaging (MRI and/or perineal ultrasound) examinations should be included in future studies to enable more accurate distinction of defect patterns. Another limitation is the retrospective nature of this study. A prospective study with postoperative urodynamic measurements should be performed to obtain more information about urethral closure. Other study limitations are related to catheter traction, which is performed subjectively according to the examiner´s judgement. Constant traction could be provided using a Newton measurer. However, the same surgeon performed all surgeries and examinations included in this study in a standardized way.

The results of this study raise various questions whose scientific examination requires prospective study performance. Future prospective studies should investigate associations of the anatomic urethral length with the SUI status after prolapse repair, including with postoperative urodynamic testing and MRI examination, as well as outcomes at various follow-up intervals. The functional impact of urethral length in the context of connective-tissue aging also warrants further examination. Our group plans to further investigate the influence of urethral length on functional aspects of urinary continence.

## Data Availability

The data that support the findings of this study are available from the corresponding author on reasonable request.

## Abbreviations

SUI: Stress urinary incontinence
ISD: Intrinsic sphincter deficiency
POP: Pelvic organ prolapse
TVT: Tension free vaginal tape
QoL: Quality of life
MRI: Magnetic resonance imaging
